# Supplementary material for: Stakeholders’ perceptions of recovery from psychosis in Blantyre, Malawi: Definitions, goals, and interventions
Source: PLOS Ment Health. 2026 Jan 27;3(1):e0000451. doi: 10.1371/journal.pmen.0000451 (PMC12843531; doi:10.1371/journal.pmen.0000451)
Supplement: Attachment — Submitted filename: Response to Reviewers.docx [file pmen.0000451.s002.docx]

Dear Editors,

*We thank you for your reviews. We have responded to the comments as requested and made all necessary changes to the manuscript in tracked changes. Our detailed responses to your comments have been italicized below*

*Sincerely,*

*Authors*

Reviewer #1 This qualitative study highlights how different stakeholders in Malawi conceptualize recovery from psychosis and also highlights an evidence-based CBD approach in a cultural context. The methodology, analysis, and interpretation are sound, and the conclusion is consistent with the data. However, minor grammatical and typographical edits are suggested, like spacing issues. Also, the study needs to clarify the theoretical framework of recovery within established models (CHIME framework) and global recovery scenarios.

*We have reviewed the manuscript and addressed all grammatical and typographical edits.*

*We have also added a paragraph in the Discussion section (7^th^ paragraph at the end of the Discussion section) that describes our results in relation to the CHIME framework*

*‘’Our findings support established conceptual models detailing processes for personal recovery among people with severe mental illness, such as CHIME (58). CHIME identifies five key components of individual recovery: connectedness, hope and optimism about the future, identity, meaning in life, and empowerment (58-61). The domain of connectedness is reflected in participants’ emphasis on the need for strong support systems among medical professionals, families and community networks as part of recovery. PWLE’s and caregivers’ aspirations for education, independent living, and future achievements also resonate with the hope component. PWLE indirectly touched on identity and meaning, through a focus on rebuilding their lives, improving their quality of life and having meaningful goals and social roles beyond their illness. PWLE and other cadres also emphasized the importance of regaining control over their lives by taking care of themselves, maintaining their independence, graduating from school, starting businesses, and establishing relationships, which align with the CHIME framework's empowerment component. While our data collection and analysis were not explicitly informed by CHIME, future research unpacking recovery could be strengthened by applying established recovery frameworks.’’*

In the result section, the statements/ quotations of participants need to be simplified.

*We have revised most quotations to simplify. However, some quotes have not been simplified as the words described by the participants allowed their thoughts and words to be fully contextualized.*

Reviewer #2:  Title: Definitions, Goals, and Interventions for Recovery in Malawi. Suggested revision to ‘stakeholders’ perceptions about recovery from psychosis in Blantyre, Malawi.’

*We have changed the title to ‘’stakeholders’ perceptions of recovery from psychosis in Blantyre, Malawi: definitions, goals and interventions’’*

1. The authors talked about the parent study but failed to provide information concerning which aspect was being presented in this particular manuscript. Please make this clear.

*We thank the reviewer for identifying this area of confusion. In our analysis, we have added the following sentences: “For this analysis, we focused on the themes that included understanding psychosis, perspectives on recovery, goals for recovery, and interventions for successful treatment.”*

2. The authors did not state the study design explicitly nor the technique for recruitment. Please make this clear. Although this was described as a hospital-based study, participants were drawn from the hospital and the community. Authors need to describe the setting better in this regard.

*We thank the reviewer for this comment. We have endeavored to more explicitly state the study design. In the parent study section, we revised. “This manuscript presents findings from* ***a qualitative study*** *conducted during the formative phase of the ENHANCE study (R34MH131234).”*

*In data collection tools and processes, we have added detail on the research methods: “The research team developed semi-structured interview guides based on a constructivist epistemology and formative research methodologies.”*

*Under the study population and recruitment, we have added detail on the recruitment procedures and setting: “religious leaders, community leaders, and traditional healers were recruited from communities that the hospital serves (i.e., within Blantyre).”*

3. The authors also need to give information on specific inclusion and exclusion criteria for their participants. For example, did patients who were recruited need to have a diagnosis for a specific length of time, or were even recently diagnosed patients included?

*Under the study population and recruitment, we have added the requested detail on inclusion for patients. “**Only patients who had the diagnosis for over 1 year, were 18 years old and above, and resided in Blantyre, were eligible.*

4. For the caregivers, did they need to live with patients or be involved in their care for a specific period to be eligible, or were these not considered? What about the time in the designated role for the practitioners? Authors need to state whether these were considered, and if not, they should consider including the information as limitations.

*We have added the following detail for caregivers: “Eligible caregivers included adults who cared for and lived with a PWLE participant.”*

*We added some additional detail with respect to the health care providers: “The lead nurse and the Head of the Department (HOD) of the clinic were asked to identify* ***mental health*** *practitioners* ***who provide care to people with psychosis*** *and might be available to participate in the interviews. Six medical practitioners were recruited from the clinic, including medical doctors specializing in psychiatry and mental health nurses****. All medical practitioners participants had been practicing in the QECH psychiatry department for at least a year****”*

5. Line 133: has both PWLE and caregivers and PWLE and guardians. Please choose one and stick with it.

*We have replaced “guardian” with “caregiver”*

6. Authors should include the ethical clearance numbers for both IRBs.

*Ethical clearance numbers have been included from both IRBs*

Result and discussion: The authors should label the quotes to give some information regarding who made the quote. Caregiver 2 does not tell me the gender, age, or level of education of the person who made the quote. These may be important when trying to discuss the results. This is the same for all the participants.

*We have added age, gender, and level of education for all quotes*

7. Line 218 mentions MP. I presume that this means medical practitioner, but the authors need to spell out each acronym before use. The phrase ‘this view may be at odds with views from MP’ is not explained as part of the result. Please consider revising or removing.

*We have spelled the acronym and removed the phrase that read “this view may be at odds with views from MP”*
